# Review: Insights into molecular mechanisms of disease in neurodegeneration with brain iron accumulation: unifying theories

**DOI:** 10.1111/nan.12242

**Published:** 2015-06-02

**Authors:** C. E. Arber, A. Li, H. Houlden, S. Wray

**Affiliations:** ^1^ Department of Molecular NeuroscienceInstitute of NeurologyUniversity College LondonLondonUK; ^2^ Reta Lila Weston InstituteInstitute of NeurologyUniversity College LondonLondonUK

**Keywords:** autophagy, mitochondria, NBIA, neurodegeneration, Tau, α‐synuclein

## Abstract

Neurodegeneration with brain iron accumulation (NBIA) is a group of disorders characterized by dystonia, parkinsonism and spasticity. Iron accumulates in the basal ganglia and may be accompanied by Lewy bodies, axonal swellings and hyperphosphorylated tau depending on NBIA subtype. Mutations in 10 genes have been associated with NBIA that include Ceruloplasmin (*Cp*) and ferritin light chain (*FTL*), both directly involved in iron homeostasis, as well as Pantothenate Kinase 2 (*PANK2*), Phospholipase A2 group 6 (*PLA2G6*), Fatty acid hydroxylase 2 (*FA2H*), Coenzyme A synthase (*COASY*), *C* *19orf12*, *WDR* *45* and *DCAF* *17* (*C* *2orf37*). These genes are involved in seemingly unrelated cellular pathways, such as lipid metabolism, Coenzyme A synthesis and autophagy. A greater understanding of the cellular pathways that link these genes and the disease mechanisms leading to iron dyshomeostasis is needed. Additionally, the major overlap seen between NBIA and more common neurodegenerative diseases may highlight conserved disease processes. In this review, we will discuss clinical and pathological findings for each NBIA‐related gene, discuss proposed disease mechanisms such as mitochondrial health, oxidative damage, autophagy/mitophagy and iron homeostasis, and speculate the potential overlap between NBIA subtypes.

## Introduction

Neurodegeneration with brain iron accumulation (NBIA) is a group of neurodegenerative diseases characterized by iron accumulation in the basal ganglia. Specifically, excess iron accumulates in the globus pallidus (GP) and the substantia nigra (SN) and can be visualized with MRI. The cortex and the cerebellum can be affected, and cerebellar involvement correlates with the most severe NBIA subtypes. The SN and the GP naturally contain high iron concentrations [1, 2] and also have a high metabolic requirement, potentially predisposing these areas to iron‐related damage.

The accumulation of iron has neither been proven to be cause nor symptom in NBIA. Iron can shuttle between two redox states and so is utilized by the cell for electron donation. ‘Free’ iron [the so called labile iron pool (LIP)] is highly reactive and can be destructive to the cell via formation of reactive oxygen species (ROS) [3]. For this reason, there are precise homeostatic mechanisms to tightly control iron levels in the cell (for central nervous system review, see Rouault [4]; for peripheral systems, see Andrews and Schmidt [5]). In the central nervous system, extracellular iron is bound by Transferrin and internalized into the cell via Transferrin receptor‐based endocytosis and the channel DMT1. Cytosolic iron is stored in Ferritin structures or transferred to organelles that are iron rich, such as mitochondria. Mitochondria have devoted iron importers and storage proteins. Cellular iron is exported via Ferroportin with the support of ferroxidases: Ceruloplasmin (Cp; mostly astrocytes) and Hephaestin (widespread). Oxidative state is controlled by ferroxidases through the pathway, such as DMT1, Ferritin and Cp. Transcription of these iron homeostasis genes is controlled closely via Iron Regulatory Protein 1/2, which control expression based on cellular iron concentration (reviewed [6]).

As well as iron accumulation, patients exhibit dystonia, parkinsonism and spasticity. Genetically confirmed pathological studies have identified protein aggregates and axonal swellings that are reminiscent of other common neurodegenerative disorders [7]. NBIA was previously known as Hallervorden‐Spatz disease if onset was after the first decade of life and infantile‐neuroaxonal dystrophy with early onset; however, genetic findings are redefining the disease.

**Figure 1:**
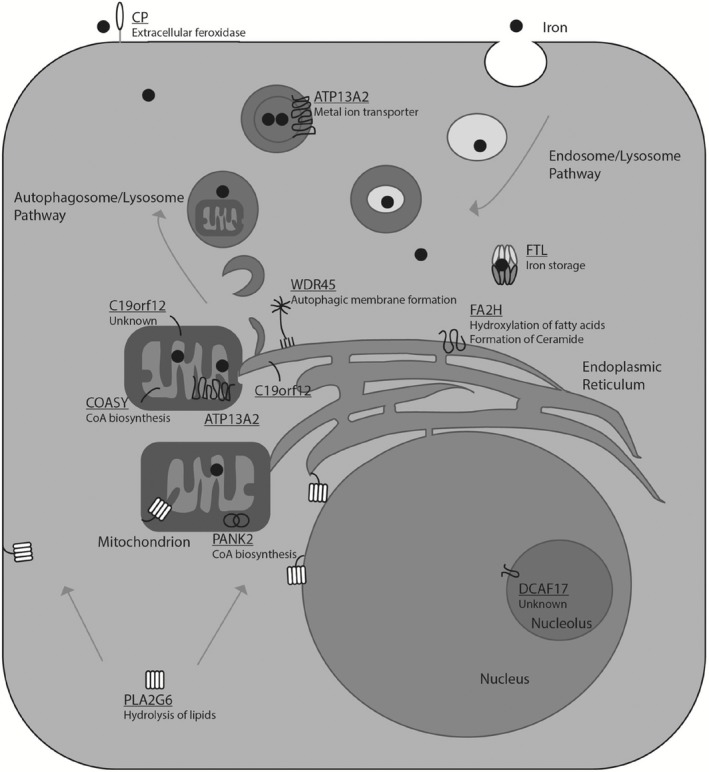
Cellular localization of NBIA‐associated genes. Iron (black circles) is taken up via transferrin‐mediated endocytosis (upper right). Cytoplasmic iron is stored in ferritin, made up of 24 monomers of ferritin light chain (FTL) and ferritin heavy chain. GPI‐anchored Ceruloplasmin (CP) facilitates Transferrin‐mediated cellular export of iron. Free CP is also present in serum. Mitochondria and lysosomes contain most of the total cellular iron. Pantothenate kinase 2 (PANK2) is a dimer and localized to intermembrane space of the mitochondria. CoA Synthase (COASY) and C19orf12 are currently believed to reside in the inner mitochondrial membrane and the outer mitochondrial membrane, respectively. COASY has a single transmembrane domain, and C19orf12 has two membrane spanning domains. Phospholipase A2 G6 (PLA2G6) is a cytoplasmic tetramer that, upon activation, can be oleoylated and associated to the plasma membrane, mitochondrial membranes, endoplasmic reticulum (ER) and nuclear envelope. WDR45 is a seven bladed β‐propeller protein that binds to phosphoinositol‐3‐phosphate‐enriched membranes at the ER. Fatty acid hydroxylase 2 (FA2H) is a four‐pass protein located in the ER. ATP13A2 is a 10‐pass polypeptide that resides in the lysosomal compartment (dark vesicles) and possibly the mitochondrial inner membrane. Finally, DCAF17 is a single pass protein located in the nucleolus.

Genetic screening over the last decade has identified 10 disease‐associated genes which lead to NBIA; however, around 20% of NBIA cases remain genetically undefined [8]. At first glance, these 10 genes appear to be unrelated and are involved in diverse cellular pathways (Figure 1). Only two genes are directly associated with iron homeostasis.

A greater understanding of the NBIA genes and any shared cellular function will ultimately help to link common clinical presentation, MRI findings and disease processes. Additionally, elucidating disease mechanisms that lead to NBIA may be relevant to other diseases such as frontotemporal dementia (FTD), Parkinson's disease (PD), Alzheimer's disease (AD), Friedreich's ataxia and amyotrophic lateral sclerosis (ALS), which display similar aspects of disease [9]. Thus, utilising the monogenetic orphan diseases is of great use to define disease processes that are involved in common and genetically undefined diseases.

The clinical features associated with the various genetic causes of NBIA are outlined in Table 1, and the pathology of NBIA is reviewed in Table 2. In this review, we will discuss potential disease mechanisms for each gene defect and finally propose potential cellular processes that link the entire NBIA spectrum.

**Table 1:** Clinical phenotype of NBIA disorders

Gene	NBIA subtype	NBIA % [8]	Associated diseases	Clinical symptoms	Onset	MRI characteristics	Reference
*PANK2*	Pantothenate kinase‐associated neurodegeneration (PKAN) (NBIA1)	35–50	HARP syndrome	Dystonia, spasticity and Parkinsonism Cognition often spared	Juvenile and adult onset (3 years or approximately 20 years)	Hypointensity with central hyperintensity of the GP, referred to as ‘eye of the tiger’	Hayflick *et al*. [10]
*COASY*	COASY protein‐associated neurodegeneration (CoPAN)	<1	–	Spasticity, dystonia, dysarthria and Parkinsonism Cognitive decline	Juvenile onset (2.5 years)	Hypointensity with central hyperintensity of the GP	Dusi *et al*. [11]
*C19orf12*	Mitochondrial membrane‐associated neurodegeneration (MPAN) (NBIA4)	6–10	SPG43	Spasticity, dystonia, dysarthria and Parkinsonism Cognitive decline	Childhood onset (11 years)	Hypointensity of the GP and SN plus hyperintensity in the GP	Hartig *et al*. [12]
*PLA2G6*	PLA2G6‐associated neurodegeneration (PLAN) (NBIA2)	20	INAD, Dystonia Parkinsonism (PARK14)	Hypotonia, spasticity, dystonia, Parkinsonism and cerebellar ataxia. Motor and mental retardation	Infantile, juvenile and late onset (1 year or 4 years or >18 years)	Hypointensity of the GP in a subset of patients Cerebellar atrophy. Some cortical atrophy	Morgan *et al*. [13]
*FA2H*	FA2H‐associated neurodegeneration (FAHN)	<1	SPG35, Leukodystrophy	Spasticity, ataxia and dystonia	Childhood onset (4 years)	Hypointensity of the GP Cerebellar and cortical atrophy	Kruer *et al*. [14]
*WDR45*	β‐propeller‐associated neurodegeneration (BPAN)	1–2	SENDA	Parkinsonism, dystonia and dementia Developmental delay, cognitive disturbances	Childhood onset	Hypointensity of the GP/SN with central hyperintense line Cerebral and cerebellar atrophy	Hayflick *et al*. [15]
*ATP13A2*	Kufor‐Rakeb syndrome	<1	Juvenile onset Parkinsonism (PARK9), Neuronal Ceroid Lipofuscinosis	Parkinsonism, dementia and some pyramidal signs	Juvenile and late onset (<20 years and <40 years)	General atrophy and hypointensity in the basal ganglia/caudo‐putamen	Schneider *et al*. [16]
*DCAF17*	Woodhouse Sakati syndrome	<1	Diabetes, alopecia, hypogonadism, deafness	Dystonia Mental retardation	Juvenile to adult onset	Hypointensity of the GP and SN	Alazami *et al*. [17]
*CP*	Aceruloplasminaemia	<1	Diabetes and anaemia	Dystonia, dyskinesia and cerebellar ataxia Cognitive impairment	Adult onset (51 years)	Hypointense striatum, thalamus and dentate	Yoshida *et al*. [18]
*FTL*	Neuroferritinopathy (NBIA3)	<1	–	Dystonia, spasticity, rigidity and Parkinsonism. Some cognitive impairment	Adult onset (39 years)	Hypointensity in basal ganglia, especially GP and SN. Also motor cortex	Curtis *et al*. [19]

**Table 2:** Pathological findings from gene‐confirmed NBIA cases

Gene	Gene confirmed pathology cases	Gross morphology findings	Iron	Axonal spheroids	Lewy body pathology	Tau pathology	Gliosis	Refs
*PANK2*	9	Neuronal loss in GP. Reduced myelin Normal SN	GP neurones, glia, microglia, macrophages, perivascular and iron dusting	GP Ubq, NF‐H, APP	No	Occasional tangles and threads	GP and widespread	Kruer *et al*. and Li *et al*. [20, 21]
*COASY*	0	Proposed similar to PANK2						
*C19orf12*	2	Neuronal loss in GP. Reduced myelin GP and SN atrophy	GP neurones, macrophages, less in astrocytes	GP Ubq Cortex, GP, SN, caudate, putamen, brain stem	Severe Lewy bodies and Lewy neurites GP, SN, cortex, striatum	Rare hyperphosphorylated Tau inclusions Cortex	Widespread	Hartig *et al*. and Hogarth *et al*. [12, 22]
*PLA2G6*	7	Cerebellar, cortical, GP and brain stem atrophy SNr particularly affected Cerebellar granule cells > purkinje cells	GP and sparse in SNr Macrophages and perivascular	Severe p‐NF, Ubq, APP, α‐syn Tubulovesicular structures GP, cortex, cerebellum, basal ganglia, striatum, brain stem	Severe, Lewy bodies and Lewy neurites Ubq, α‐syn SNc, cortex	Early onset hyper phosphorylated Tau inclusions, threads and tangles Cortex	Variable	Gregory *et al*. and Paisán‐Ruiz *et al*. [23, 24, 25]
*FA2H*	0	Proposed brainstem atrophy and demyelination		Proposed white matter lesions and enlarged axons				
*WDR45*	1	SN > GP neuronal loss Cerebellar atrophy, purkinje and granule layer, cortical atrophy	Strongest in SN Also GP and glia	GP, SN plus thalamus	No	Tau tangles, hippocampus, cortex, putamen, few in atrophied SN and GP	Putamen and thalamus	Hayflick *et al*. [15]
*ATP13A2*	0			Peripheral biopsies show demyelination and cytoplasmic inclusions in nerve and muscle tissue				
*DCAF17*	0			Peripheral biopsies show denervation of muscle tissue				
*CP*	6	Severe: putamen, dentate nucleus. Moderate: GP, cerebellum purkinje; mild: SN, cortex Neuronal atrophy Iron in visceral organs	Similar to neurone loss, cerebellum, GP > SN, cortex Perivascular and astrocytic terminals	Iron laden ‘globular structures’ present in glia and variable in neurones	Unknown	Unknown	Yes	Gonzalez‐Cuyar *et al*., Kaneko *et al*., Morita *et al*., Oide *et al*. [26, 27, 28, 29]
*FTL*	4	Mild atrophy in the cerebellum, cortex, putamen Putamen and cerebellum most affected GPe and SN mildly affected	Cerebellum and putamen Glia, perivascular and perineuronal	Yes, GP, Ubq and NF Ferritin and iron laden inclusions in glia > neurones GP, putamen, thalamus and cerebellar cortex	Unknown	Few	Yes, but some atrophy too	Curtis *et al*., Vidal *et al*., Mancuso *et al*. [19, 30, 31]

APP, amyloid precursor protein; GPe, globus pallidus externa; GPi, globus pallidus interna; NF, neurofilament; SNc, substantia nigra pars compacta; SNr, substantia nigra pars reticulata; Ubq, ubiquitin; α‐syn, alpha synuclein.

## PANK2 and PKAN

Mutations in the Pantothenate Kinase 2 gene (*PANK2*) lead to Pantothenate Kinase‐associated neurodegeneration (PKAN, NBIA type 1). PKAN represents the most common genetic cause of NBIA and occurs in around two‐thirds of NBIA patients [10, 32].

*PANK2* is a seven exon gene which is alternatively spliced to form two transcript variants [33]. Mutations have been described throughout the gene leading to autosomal recessive PKAN. There is a strong correlation between loss of enzymatic activity and disease onset, for example, two null mutations will present with an early‐onset phenotype [34, 35]. Despite the fact that PANK2 proteins form homodimers, it is unlikely that mutant PANK2 has a dominant negative effect, as co‐expression of mutant PANK2 and wild‐type PANK2 shows similar activity to wild‐type PANK2 alone [33]. The most frequent mutation is G521R which is thought to contribute to 25% of the disease alleles [10], and this mutant protein fails to fold properly, exhibiting no enzymatic activity [33].

PANK2 is one of four human pantothenate kinase proteins and is specifically located in the mitochondria [36, 37]. PANK4 is proposed to be nonfunctional while PANK1 and PANK3 are active in the cytosol. The PANK2 polypeptide contains a mitochondrial localization signal. There is a catalytic ATP binding domain located at the N‐terminus of the protein and a domain of unknown function at the C‐terminus of the protein. PANK2 homodimers are located in the mitochondrial intermembrane space.

### Pathology

Nine confirmed PKAN patients have been studied *post mortem*, exhibiting predominant GP pathology (Table 2) [20, 21]. In all cases, the GP was discoloured and showed excess iron accumulation. Iron was distributed in degenerating neurones, macrophages, astrocytes, microglia and also in perivascular regions. Neuronal cell loss was evident and largely restricted to the GP, together with associated axonal loss. Astrogliosis of the GP was a common feature.

Cortical and subcortical brain regions show axonal swellings and spheroid bodies. These represent degenerating neurones and dystrophic axons. Swellings are immunoreactive at varying degrees for ubiquitin, amyloid precursor protein and phosphorylated neurofilament [20, 21]. A loss of myelin and vacuolization has been described in the pallidum [21], potentially linking PKAN disease processes to other demyelinating conditions.

Tau pathology has been noted to a minor extent in all cases, with tangles and threads present in the cortex [21]. Historically, NBIA was thought to be an alpha‐synucleinopathy; however, genetic screening methods have been distinguished PKAN, which shows no α‐synuclein accumulation, with other NBIA subtypes that do, for example MPAN and PLAN (see below).

### PANK2 mechanisms

The primary function of PANK is to catalyse the ATP‐dependent phosphorylation of dietary pantothenate (vitamin b5) to 4‐phosphopantethenate, the first step in Coenzyme A (CoA) biosynthesis. CoA is central to metabolism and is required for β‐oxidation, the citric acid cycle and the metabolism of amino acids and ketone bodies. CoA is also required for synthesis of fatty acids and amino acids.

A prominent hypothesis for neurodegeneration caused by PANK2 deficiency is that the lack of active enzyme leads to build‐up of substrates in the CoA biosynthetic pathway. This leads to an accumulation of N‐pantothenyl cysteine and free cysteine. Cysteine build‐up can chelate iron and lead to its accumulation. Free cysteine can auto‐oxidize in the presence of iron and produce ROS, resulting in widespread oxidative damage and cell death, perhaps through lipid peroxidation [32, 38]. Iron content is naturally high in the GP and SN [1], potentially sparking degeneration synergistically with excess cysteine, followed by a negative feedback of iron accumulation and ROS‐derived damage.

The localization of PANK2 at the mitochondria (Figure 1) prompted several groups to investigate mitochondrial dysfunction in PKAN pathogenesis. *PANK2* mutant fibroblasts [39] as well as mouse and fly *pank* knockout models [40, 41, 42] have hinted to mitochondrial deficiencies, including reduced mitochondrial membrane potential, swollen mitochondria with altered cristae and ruptured membranes. ROS damage has been described with varying degrees.

The loss of mitochondrial membrane potential can affect the rate of mitophagy, and it is feasible that altered mitochondrial fission and/or trafficking, together with membrane remodelling deficiencies due to lower levels of CoA and acyl‐CoA, might contribute to axonal swellings [43, 44].

*Drosophila* have one pantothenate kinase, *fumbl.* The *fumbl* hypomorphic model displays locomotor defects and lipid dyshomeostasis which are fully rescued by human PANK2 [40]. This highlights the high degree of protein conservation between species [45]. Importantly, human PANK3 and PANK4, which are cytosolic proteins, cannot fully rescue drosophila mutants, hinting to a cellular dependence on specific PANK2 activity [45].

There is a rate limiting feedback inhibition step by acyl‐CoAs on the ATP binding pocket of PANK2 [34]. The negative regulation of PANK2 by acetyl‐CoA is so tight that mechanisms are in place to relieve this inhibition. For example, palmitoylcarnitine activates PANK2 in physiological conditions [46]. Enzymatic regulatory deficiencies could thus be involved in disease. However, three mutant proteins tested *in vitro* display similar acetyl‐CoA inhibition [33].

PANK2 may have a role in sensing CoA concentration and the metabolic cross‐talk between the mitochondria and the cytoplasm [46]. This could explain why genetic mutations not affecting enzymatic activity could lead to a disease phenotype. In addition, pantothenate kinase‐derived CoA is responsible for acetylation of proteins [47]. Protein acetylation and CoA levels have been shown to directly link cellular metabolism and autophagic flux [48, 49]. This implicates a new pathway that is distinct from the well‐documented processes controlling autophagy, including AMPK and mTOR1.

PANK2 mutations could disrupt a putative mitochondrial‐specific fatty acid synthase pathway – critical for mitochondrial membrane assembly and function [37]. One study has confirmed lipid dyshomeostasis via a metabolomics approach on blood plasma of PKAN patients [50]. Furthermore, the clinical overlap described between PKAN and HARP syndrome (hypoprebetalipoproteinaemia, acanthocytosis, retinitis pigmentosa and pallidal degeneration), which both derive from mutations in *PANK2*, strongly links lipid production deficits in *PANK2* mutants [51].

It is important to consider the role of excess iron in PKAN. PANK2 is targeted to the mitochondria, which are a major sink of iron within the cells, and multiple proteins involved in the electron transport chain contain iron‐sulphur clusters. Knockdown of PANK2 in HELA cells and hepatocytes leads to a marked induction of ferroportin mRNA, the sole cellular exporter of iron [52]. Additionally, patient fibroblasts showed a reduced response to excess iron, with reduced expression of ferritin (the cellular iron storage protein) and an increased LIP [39]. Increased labile iron could lead to ROS directly, due to the Fenton reaction, whereby water is broken down in the presence of iron to form ROS.

In summary, PKAN patients display excess iron deposition and neuronal cell death, primarily in the GP. *PANK2* mutations might affect metabolism and lipid turnover and ultimate affect energy production or mitochondrial fitness. Conversely, reduced CoA levels have not been proven in patient tissue, and so, disease mechanisms through alternate unknown PANK2 functions remain a possibility. Investigations into cellular mechanisms of cell death are required to understand the reasons for specific degeneration due to this gene defect.

## COASY and CoPAN

Mutations in the CoA Synthase (*COASY*) gene were recently shown to lead to NBIA [11]. Clinically, COASY protein‐associated neurodegeneration (CoPAN) patients display similar clinical features to PKAN patients [11].

The COASY protein contains a mitochondrial localization signal, a regulatory region and a domain for each of the two catalytic kinase domains: adenyl transferase and dephospho CoA. COASY is localized to the mitochondrial matrix [11, 53]. There are two COASY isoforms, produced from alternate splicing. The longer β isoform is brain specific and has an additional proline‐rich protein interaction domain but has identical enzymatic activity to the ubiquitous α isoform [54].

Two CoPAN mutations described by Dusi and colleagues represent a premature stop codon (G49Stop) and a mutation affecting a conserved residue involved in ATP binding and dephospho‐CoA kinase activity (R499C). These are proposed to completely abrogate the protein functionality [55].

Interestingly, yeast knockout models for the two proteins that are COASY orthologues lead to a lethal phenotype. Human wild‐type COASY protein can rescue this lethality, and mutant COASY from patients can rescue knockout cells but lead to a higher requirement for pantothenate in the growth media [11].

### COASY mechanisms

COASY is in the same metabolic pathway as PANK2 and is a bifunctional enzyme catalysing the final two steps of CoA synthesis [53, 56]. Mutant COASY protein appears to have no activity *in vitro*, whereas CoA levels in patient and control fibroblasts appear normal [11]. This suggests CoA levels are maintained by either residual activity of the COASY protein, or via an alternative, as yet unknown, pathway for CoA synthesis.

The fact that COASY acts in the same pathway as PANK2 suggests common mechanisms may be shared between PKAN and COPAN, such as reduced acyl‐CoA and lipid synthesis leading to mitochondrial insufficiencies.

Of the 10 NBIA‐related genes, only *COASY* shows a putative iron response element (IRE) via the SIREs 2.0 prediction tool (http://ccbg.imppc.org/sires/). The sequence is a putative 3′ IRE and would lead to mRNA stabilization in the presence of iron. This could potentially link *COASY* expression to iron availability in the cell.

## C19orf12 and MPAN

C19orf12 is a protein located on the outer mitochondrial membrane with unknown function (Figure 1). Mutations in *C19orf12* lead to mitochondrial membrane protein‐associated neurodegeneration (MPAN). MPAN accounts for around 30% of NBIA [22]. Other diseases associated with *C19orf12* mutations include pallido‐pyramidal syndrome [57], hereditary spastic paraplegia type 43 (SPG43) [58] and ALS [59].

C19orf12 is a two‐pass transmembrane protein with two alternative start codons. Missense mutations have been described to add charged amino acids to putative hairpin domains, potentially disrupting the 3D structure [12, 58]. MPAN has been successfully modelled in drosophila [60].

Post‐mortem examination of two MPAN cases [12, 22] showed iron accumulation mainly confined to the GP. Iron accumulation was accompanied by neuronal atrophy, and depositions were present in neurones, macrophages and, to a lesser extent, astrocytes (Table 2).

Lewy bodies and Lewy neurites were a characteristic feature of MPAN with an extremely high burden [22]. Lewy neurites in the GP were adjacent to the atrophied region and Lewy pathology extended to the SN where they were associated with near complete neuronal loss. Lewy bodies were also present in the cortex, striatum and hippocampus. Axonal swellings and hyperphosphorylated tau were also present in the cortex, GP, caudate/putamen, SN and brain stem. Demyelination was evident in the spinal pyramidal tracts and optic nerve.

### C19orf12 mechanisms

C19orf12 may be expressed in the ER as well as the mitochondria [58], and pathogenic mutations that lead to SPG43 or MPAN alter the distribution of the protein, potentially hinting to a lack of proper protein folding and enzymatic activity. C19orf12 is expressed in neurones, white blood cells and adipocytes [60]. During *in vitro* differentiation of white blood cells, C19orf12 expression closely follows fatty acid metabolism, suggesting a link to lipid homeostasis [12]. It was further proposed that this function could be associated with CoA metabolism, linking MPAN to PKAN and CoPAN [12].

Therefore, C19orf12, PANK2 and COASY are linked through mitochondrial localization, lipid metabolism and ultimately iron deposition and Tau pathology in disease.

## PLA2G6 and PLAN

Mutations in the gene encoding phospholipase A_2_ group VI calcium‐independent (*PLA2G6*, also known as *iPLA_2_β* or *iPLA_2_‐VIA*) have been associated with two diseases: phospholipase associated neurodegeneration (PLAN, NBIA type 2) and dystonia‐parkinsonism (Table 1). PLA2G6‐associated disease may have previously represented infantile neuroaxonal dystrophy (INAD) before NBIA reclassification.

There are four groups of PLA2s containing more than 20 proteins: secreted PLA2s, calcium‐dependent cytosolic PLA2s, platelet‐activating acetylhydrolases and calcium‐independent PLA2s (iPLA2) [61]. PLA2G6 is responsible for 70% of total PLA2 activity in the brain [62].

The *PLA2G6* gene encodes an 806 amino acid protein with predicted size of 88 kDa. The mature protein is tetrameric and composed of an ankyrin repeat domain that is involved in protein interaction, a GXSXG lipase motif, an ATP binding pocket and a Calmodulin binding domain [61]. There are at least five splice variants, three of which contain the lipase motif GXSXG, whereas two contain only the ankyrin repeats and most likely act as competitive inhibitors within the tetramer [63].

PLA2G6 has been shown to be expressed in the mitochondria [64, 65] and be protective to mitochondrial health [66]. Expression is also seen at the nuclear envelope and at axon terminals in primate brain tissue [67]. PLA2G6 is ubiquitously expressed [68], although disease symptoms are solely neurological.

Autosomal recessive mutations are found throughout *PLA2G6* and lead to reduced enzyme activity [13]. The magnitude of effect on enzyme activity correlates with disease severity: two null copies of PlA2G6 have the most severe phenotype [23]. Dystonia‐parkinsonism mutations do not affect the enzymatic ability of the protein but could alter protein interactions [69].

The enzyme can undergo autoacylation by oleoyl‐CoA near the catalytic residue [70]. Acylation leads to a cytosolic to membrane relocation of PLA2G6 and may lead to precise subcellular targeting of the enzyme and potential catalytic activation (Figure 1).

### Pathology

The pathology of seven confirmed PLAN cases have been studied (Table 2). The major pathological hallmark of PLAN is the presence of axonal swellings throughout the cortex, GP, striatum, cerebellum, brain stem and spinal cord. These are also present throughout the peripheral nervous system and can be diagnostic via peripheral nerve biopsies. There is evidence for *PLA2G6* mutation‐associated PLAN without spheroids [13]. Peripheral spheroids are not observed in dystonia‐parkinsonism subset of PLAN patients [71].

Spheroids stain positive for phosphorylated neurofilament, ubiquitin and APP. α‐Synuclein positive Lewy bodies are found throughout the degenerating brain areas to a very high degree, and a proportion of the axonal swellings also stained positive for α‐Syn. Lewy bodies were specifically seen in the SN pars compacta. Cases also exhibited advanced tau pathology in early adulthood [23, 24, 25], whereby braak stage V/VI neurofibrillary tangles and neuropil threads were evident in cortical areas at the age of 18 and 32 [25]. Granules within spheroids of *pla2g6* mutant mice were positive for Periodic‐acid Schiff (PAS) staining. This is likely to represent polysaccharides, glycated proteins or aldehyde products of ROS‐damaged lipids [72].

Tubulovesicular structures, another characteristic of PLAN, are membrane‐rich inclusions and thought to contain mitochondria, lysosomal components, vacuoles, endoplasmic reticulum as well as occasionally a protein component [73].

Atrophy is evident in the cortex, GP, white matter, the cerebellar granule cells and cerebellar purkinje cells and was shown to be associated with gliosis in the cortex and the striatum [25]. Excess iron deposition was specifically observed in the GP and the SN pars reticulata [23]. There is extensive cortical involvement in disease, and Lewy bodies are seen in all cases whereas tau deposition is rarer [24, 25].

### PLA2G6 mechanisms

PLA2G6 hydrolyses glycerophospholipids at the sn‐2 position of acyl chains to produce lysophospholipids and free fatty acids, such as arachidonic acid. The downstream metabolites of free fatty acids such as leukotrienes and prostaglandin perform essential cellular functions and contribute to a variety of signalling events including membrane remodelling, fatty acid oxidation, cell signalling and growth, and apoptosis [61]. The 2‐lysophophospholipid may also play a role in signalling such as in the production of platelet activating factor. Phospholipase activity is crucial for membrane integrity via phospholipid recycling and homeostasis.

PLA2G6‐mediated neurodegeneration may occur via the inability to remodel oxidized and damaged phospholipids. Polyunsaturated mitochondrial components, such as cardiolipin, are extremely sensitive to ROS. PLA2G6 has an increased affinity to membranes in hydrogen peroxide‐treated cells resulting in a raised activity and increased free fatty acid release [74]. Cells that aberrantly produce ROS sequester PLA2G6 to the mitochondria, and this is protective against apoptosis [66]. Patient cells were shown to display faulty mitochondrial at the ultrastructural level [75, 76].

Uncoupling of the mitochondrial respiratory chain and associated depolarization (e.g. in de‐energized states or after calcium ion influx) can lead to activation of PLA2G6 within the mitochondria and therefore free fatty acid accumulation [77]. This can initiate apoptosis through stimulating the permeability transition pore and cytochrome C release leading to further lipid damage and apoptotic signals [65, 78]. Reduced PLA2G6 activity may lead to dysregulation of this process.

Related to this, ER stress can increase the expression and activity of PLA2G6, bringing about the loss of mitochondrial membrane potential and increasing the likelihood of apoptosis [79].

PLA2G6 plays an important role in membrane homeostasis. The lipid profile of spinal cords of Pla2g6 knockout mice is greatly altered suggesting a defective remodelling ability. This may be causal for degenerative inner mitochondrial membranes and axonal termini [80]. Altered axonal and/or organellar membrane integrity may lead to improper axonal transport and accumulation of cellular components at distal axon locations, subsequently leading to axonal blockage and degeneration [81].

Both products of PLA2G6 catalysis, lysophospholipids and free fatty acids, may be able to increase membrane fusion via increased fluidity and nonbilayer structures in the local environment [61]. This could have important roles in autophagy, mitophagy and other vesicle‐based processes that have been shown to be involved in neurodegeneration.

Using *pla2g6* knockout mice, Beck and colleagues proposed that the disease progresses via two concurrent mechanisms [80]. Firstly, mitochondria exhibit degenerative inner membranes, and a large component of swellings was shown to be mitochondria. Faulty mitochondria transported through the axons may release ROS and proapoptotic factors that damage neighbouring membranes, producing axonal swellings. Degenerative mitochondria are accompanied by local cytoskeletal disappearance, exacerbating mitochondrial transport deficiencies. Secondly, at axon terminals, membranes and synaptic vesicles degenerate and form swellings and tubulovesicular structures. Together, the lack of membrane remodelling is proposed as the cause of the disease phenotypes seen within axons and at axon terminals, two sites where PLA2G6 is localized [66, 67].

Finally, there is evidence for a noncell autonomous effect of PLA2G6 disruption. Docosahexaenoic acid (DHA) is an essential fatty acid that cannot be synthesized in neurones. Astrocytes have been shown to produce and release DHA due to PLA2G6 action [82]. Knockdown of the enzyme in astrocytes leads to a reduction of arachidonic acid and DHA in neurones and increased prostaglandin production [83], which could lead to increased apoptosis [84].

Calcium signalling was shown to be defective in astrocytes from *pla2g6* mutant mice and in astrocytes treated with a PLA2G6 inhibitor [85]. This suggests that cross‐talk between neurones and glia may be disrupted in PLAN/INAD.

*PLA2G6* is classified as a PD gene (PARK14), and also, a reduction in PLA2G6 protein levels has been shown in the brains of Alzheimer's patients [86]. Intriguingly, the SN shows low endogenous levels of PLA2G6, possibly showing a vulnerability to lipid oxidation [87]. Mitochondrial involvement, lipid turnover and Tau pathology are again implicated in this NBIA subtype.

## FA2H and FAHN

Fatty acid 2 hydroxylase (*FA2H*) mutations are linked to leukodystrophy, hereditary spastic paraplegia (*SPG35*) and NBIA [14, 88, 89].

FAHN is a recessive disease, and it has been shown for SPG35‐associated mutations that the enzyme is nonfunctional [88]. FA2H is a 43 kDa membrane‐bound protein, residing in the ER [90]. The polypeptide has a C terminal sterol desaturase domain, which contains an iron binding histidine motif and is responsible for catalytic activity, and an N terminal cytochrome b_5_ haem‐binding domain, involved in redox activity and electron donation to the C terminus [91, 92].

Mouse knockout models provide a hint towards FAHN pathology, whereby the CNS exhibits enlarged axons and demyelination [93]; however, no human post‐mortem studies have been reported.

### FA2H mechanisms

FA2H is an enzyme that catalyses the hydroxylation of fatty acids at position 2 of the N‐acyl chain (Figure 1). 2‐Hydroxy‐fatty acids are a precursor for Ceramide synthesis, a critical component of myelin sheaths [90]. Inactivating mutations in *FA2H* is likely to affect normal myelin production due to loss of the hydroxylase activity of the enzyme. The late onset and slow progression are consistent with this idea, analogous to demyelinating diseases such as multiple sclerosis (MS).

Altered ceramide signalling may have roles in Lewy body formation, and the role of ceramide in apoptosis and neurodegeneration has been implicated [94, 95]. Myelin formation via FA2H is dependent on lysosomal acid ceramidase and also fatty acid oxidation in peroxisomes, potentially linking cellular compartments that are common between NBIA genetic disorders.

FA2H also has a role in lipid signalling pathways that can affect cell cycle and apoptotic pathways [92].

Iron storing Ferritin has been shown to associate with myelin [96], and it was postulated that iron accumulation in disease could be associated with faulty myelin affecting Ferritin dynamics [14].

Axonal myelination may be a common factor between NBIA subtypes. *PANK2* and *COASY* are both involved in CoA synthesis. CoA has many diverse cellular roles but is critical for the generation of sphingolipids, another principle component of myelin [50]. Defective myelination may contribute to neuronal dysfunction and apoptosis and is shared between several neurodegenerative diseases such as MS.

## WDR45 and BPAN

Mutations in the WD repeat domain 45 gene (*WDR45*) were linked to a form of NBIA and termed β‐propeller‐associated neurodegeneration (BPAN) [97].

Despite *WDR45* being on the X chromosome, BPAN does not follow normal X‐linked dominant inheritance. Both genders have similar clinical features, and the disease is always sporadic. Disease‐associated mutations are predicted to lead to nonfunctional proteins, and are presumed to be lethal for male embryos. Male BPAN sufferers are presumed to have de novo mutations and were shown to have somatic or germline mosaicism [97].

One confirmed BPAN patient has been studied *post mortem* (Table 2) [15]. Iron deposition was strongest in the SN but also evident in the GP, concurrent with cerebral and cerebellar atrophy (purkinje and granule cells). Axonal spheroids were present in the basal ganglia (especially the GP and the SN), and gliosis was prominent.

Tau‐positive neurofibrillary tangles were common in the neocortex, putamen and the hippocampus, and less common in the atrophied GP and SN. Amyloid‐β plaques and Lewy bodies were not evident [15]. Pathological changes appear to affect the SN preferentially over the GP.

### WDR45 mechanisms

WDR45 (also known as WIPI4) is a β‐propeller scaffold protein that has been predicted to have a role in autophagy. WDR45 is a member of the WD40 protein family, which provide a basis for protein–protein interactions and perform cellular functions such as autophagy, cell cycle progression and transcriptional control. WDR45 binds to phospholipids and autophagy‐related proteins [98].

The functions of WDR45 have been investigated using several models. Yeast have over 30 autophagy‐related genes (*atg*), of which many mammalian homologues have been found. WDR45 is one of 4 *atg18* homologues, critical for autophagosome formation (WIPI1‐4) [99]. *atg18* binds the ER membrane via a phosphotidylinositide‐3‐phosphate binding motif and facilitates downstream protein complex formation [100] (Figure 1).

Phosphatidylinositol‐3‐phosphate (PI3P) is a critical factor in autophagy and is a major component of autophagosome membranes. Upstream signalling (e.g. mTOR) controls PI3P and autophagic flux. PI3P may be involved in early membrane curvature and autophagosome size. Therefore, WDR45 could regulate autophagosome size and maturation [99]. The *Caenorhabditis elegans* homologue of WDR45, epg‐6, was shown to be required for regulating early autophagosome size and interacts with several other ATG‐like genes [98]. Epg‐6 recruits *atg9* which is thought to supply lipids for newly forming autophagosomes [101].

Lymphoid cells from five patients with truncated or destabilized WDR45 protein show a blockage in autophagic flux when exposed to inhibitors or activators of autophagy [102].

*atg* Molecules are transiently present on mitochondrial outer membranes suggesting a link between WDR45 autophagosomes and mitochondrial function [103]. H_2_O_2_ and ROS generated in mitochondria perform an essential role in oxidising and inactivating *atg4* so that the autophagosome may form [104], leading Scherz‐Shouval and Elazar to hypothesize a signalling gradient that allows autophagy biogenesis in the vicinity of mitochondria.

MRI scans, from patients with early disease stage presentation, suggest that iron accumulation is observed in the SN early in disease progression, and GP iron accumulation is a later event [15]. This is in contrast to other NBIA subtypes.

Finally, genes involved in autophagy are disrupted in other disease conditions. PD, Crohn's disease, cancer and spastic paraperesis have all been shown to have disrupted autophagy [105]. The pathology of BPAN and the strong involvement of the SN potentially support a shared mechanism to PD.

## ATP13A2 and Kufor‐Rakeb Syndrome

Mutations in *ATP13A2* lead to Kufor‐Rakeb Syndrome, which is a disease exhibiting juvenile onset parkinsonism and dementia (PARK9) [106], neuronal Ceroid‐Lipofuscinosis [107] and NBIA [16].

Mutations in *ATP13A2* are autosomal recessive and cluster on the cytoplasmic domains, interfering with catalytic activity [108]. Transmembrane stretches may also be affected and consequently the protein architecture.

There are no post‐mortem studies of ATP13A2 mutation‐associated NBIA. However, studies of sporadic PD patients have shown that ATP13A2 staining is increased in cortical and nigral neurones [108, 109], and ATP13A2 protein is found associated with Lewy bodies [110]. Post‐mortem studies of Kufor‐Rakeb Syndrome patients confirm the atrophy seen with MRI and also lipofuscinosis in neurones and glia of the cortex, basal ganglia and cerebellum [107].

### ATP13A2 mechanisms

ATP13A2 is a lysosomal P‐type ATPase that functions as divalent cation transporter. P‐type ATPases are a large family of transporters that also include calcium pumps, proton pumps and phospholipid flipases. This superfamily consists of highly conserved, 10‐pass transmembrane proteins, which utilize the energy stored in ATP to transport ions across membranes [111].

ATP13A2 was shown to be associated with membranes of the lysosome [108] (Figure 1), although it has also been linked with mitochondrial and synaptic membranes [109]. Knocking down the expression of *ATP13A2* affects the size and number of autophagosomes [109]. Mutations in *ATP13A2* may cause a mislocalization of the protein to the ER [112].

The overexpression of ATP13A2 in model organisms has been shown to protect against potentially cytotoxic environments such as α‐synuclein overexpression [112, 113] and heavy metal ions including cadmium, manganese, nickel and selenium [114]. Indeed, patient fibroblasts from *ATP13A2* mutants displayed lysosomal deficiencies that lead to cytotoxic effects in the presence of α‐synuclein [115] and zinc [116]. Intracellular manganese levels were shown to be higher in cells with mutant *ATP13A2* compared with wild type, hinting to a reduced secretion ability [112]. This may directly lead to cytochrome C release from mitochondria and apoptosis.

The increased cytosolic heavy metal status of the cell may be linked with the characteristic fragmented mitochondrial phenotype witnessed in mutant cells [109, 117]. Patient‐derived fibroblasts and olfactory neurones have exhibited fragmented mitochondria, reduced ATP production, oxidative stress and mitochondrial DNA lesions [118] that may be in part due to reduced intracellular free zinc levels and a sensitivity to environmental zinc [117]. Transcription of zinc homeostatic genes is upregulated in a compensatory manner.

Metal ions are enriched in acidic lysosomal compartments and could be involved in destructive conditions required for degradation and recycling events [119]. Additionally, divalent metal ion transporters are unfaithful and appear to function to transport a range of metal ions. Although iron has not been shown to be directly dysregulated in ATP13A2 deficient cells, it will be interesting to investigate this further due to the accumulation seen in the putamen of Kufor‐Rakeb patients.

**Figure 2:**
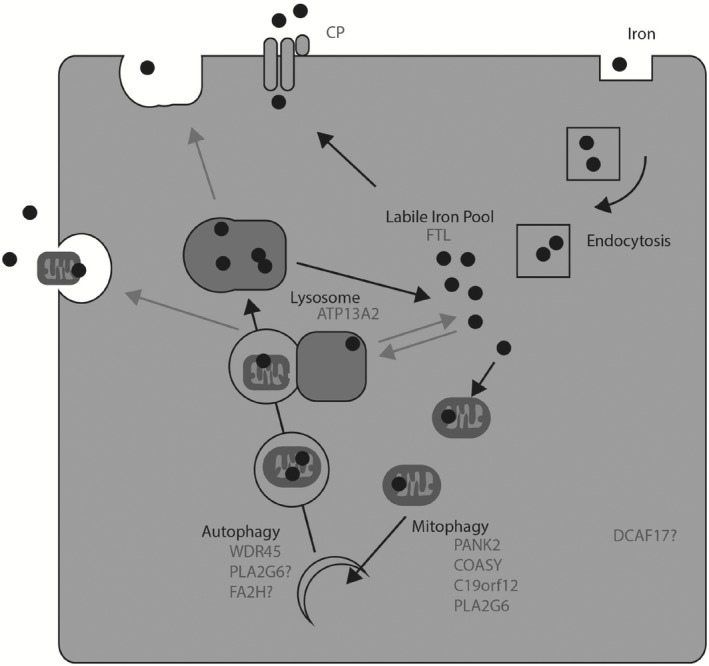
Cellular iron homeostasis and putative involvement of NBIA‐associated genes. Iron is taken up via transferrin‐mediated endocytosis and a theorized cytoplasmic pool of free iron is termed the LIP. Free iron is safely stored within Ferritin macromolecules, via Ferritin heavy chain and FTH. Cellular iron export is facilitated via the ferroxidase activity of Ceruloplasmin. High iron concentration is required in mitochondria; however, no mitochondrial iron exporter has been found. Mitophagy is one potential mechanism for iron recycling, and several NBIA‐related genes may alter the rate of mitophagy, PANK2, COASY, C19orf12 and PLA2G6. Autophagy and mitophagy are intrinsically related, and WDR45 mutations may alter the rate of these recycling processes. FA2H and PLA2G6 may impinge on autophagy via membrane remodelling and vesicle formation. ATP13A2 has a role in divalent ion transport into the lysosome compartment, and this could include/affect iron transport into the iron‐rich lysosomal compartment. DCAF17 has not been linked to iron homeostasis. Mitochondrial‐rich vesicles have been described to be exocytosed and, together with theorized lysosomal‐derived exosomes, may represent another cellular iron secretory mechanism. Grey arrows represent theorized steps in the pathway. Black circles represent iron.

Another P‐type ATPase, ATP7B, is mutated in Wilson's disease. Upon elevation of cytoplasmic copper levels, wild‐type ATP7B translocates from the golgi to the lysosome whereby it aids the loading of copper into lysosomes. Copper‐rich lysosomes are then secreted from the cell via lysosomal exocytosis [120]. Wilson's disease results from dysfunctional ATP7B, increased copper levels and altered redox state. Therefore, the faulty ATP13A2 protein may reduce a noncanonical cellular excretion mechanism for heavy metals (Figure 2).

Post‐mortem studies of NBIA‐associated ATP13A2 patient brains have not been described but will be informative with regard to pathological hallmarks. The fact that ATP13A2 is a lysosomal disorder means that there are tantalising links to lysosomal storage diseases. Additionally, patients exhibit dementia and cortical atrophy, and iron accumulation in the caudate and the putamen, hinting to ties with FTD and HD. Similar to other NBIA subtypes, there is an effect on mitochondrial health.

## DCAF17

*DCAF17* (also called *C2orf37*) is a protein of unknown function that is expressed in the nucleolus (Figure 1). Mutations in this gene lead to Woodhouse‐Sakati syndrome (WSS), and a subset of patients displays brain iron accumulation [17]. Many patients display no iron accumulation; however, the GP and the SN are affected in some patients [8].

WSS manifests during adolescence and presents with extrapyramidal symptoms, dystonia and cognitive decline. Typically, patients exhibit hypogonadism, alopecia and diabetes mellitus.

Very little is known about the function of DCAF17; however, the clinical phenotype of the patients and the expression data are very similar to the ribosomal synthesis deficiencies of patients with RBM28 mutations [121].

DCAF17 is a multipass transmembrane protein that is named due to protein–protein interactions, Ddb1 and Cul4‐associated factor (damaged DNA binding protein 1 and cullin 4 ubiquitin ligase complex) [122]. This association links DCAF17 to protein ubiquitination involved in DNA damage and cell cycle control. The nucleolar localization leads to intriguing questions about specific function and any links to other NBIA‐linked mutations.

### Iron

Many transition metal ions are critical cellular components. Iron is an essential dietary component and acts as a crucial cofactor for enzymes and proteins. Iron is synthesized into iron‐sulphur clusters and haem groups to perform catalytic events, and for example, 12 iron‐sulphur clusters and seven haem groups are required for the mitochondrial respiratory chain.

Iron is present in two oxidative forms, ferrous Fe(II) and ferric Fe(III), enabling effective electron transport. Iron that is not bound into iron‐sulphur clusters or haem groups is termed the theoretical LIP. Free ferrous iron can be extremely reactive, producing hydroxyl radicals and leading to oxidative damage. Therefore, there exists tight control over iron metabolism within the cell, whereby entry, exit, redox state and total iron content are tightly controlled (for review, see Rouault [4]). Haem consists of an iron atom at the centre of an organic porphyrin ring and has a distinct cellular metabolic pathway.

Two forms of NBIA are a result of mutations to proteins directly involved in cellular iron homeostasis, suggesting that iron metabolism defects might be causative for NBIA.

Importantly, the GP and the SN are naturally rich in iron, and iron content increases with age [1, 2]. This may predispose these brain regions to iron‐induced damage in NBIA disorders.

## Cp and aceruloplasminaemia

Cp is involved in cellular iron export. Cp is a glycoprotein containing many copper atoms (and potentially iron atoms) that acts as a ferroxidase to facilitate ferroportin‐mediated cellular iron export (Figure 1). Oxidation of Fe(II) to Fe(III) enables binding to transferrin for transport in the extracellular environment. Aceruloplasminaemia, leading to brain iron accumulation, has been shown to be associated with a lack of ferroxidase activity (reviewed [123]); however, mutant Cp protein accumulation has also been described [124].

Disease is thought to progress as a lack of Cp‐based ferroxidase activity leads to reduced cellular export of iron as well as an accumulation of extracellular transferrin‐free Fe(II) [125]. Astrocytic sequestration of nontransferrin bound extracellular iron could lead to a dearth of iron available for neurones and subsequent cell death [126]. Importantly, other cell types such as astrocytes contain an alternative export‐associated ferroxidase, Hephaestin, and may explain specific regional degeneration in aceruloplasminaemia [127].

Aceruloplasminaemia patients display increased serum nontransferrin‐bound iron, MRI‐based evidence of iron accumulation in the basal ganglia, movement disorders and dementia. Pathology includes iron deposition in neurones and glia within the basal ganglia and dentate nucleus (Table 2) [26, 27, 28]. The retina and cerebellum may also show iron accumulation as well as the cortex in late stage disease. Astrocytes show spheroid‐like globular structures and iron‐rich inclusions in their processes [26]. Oxidative damage was shown to be increased in patient tissue, possibly due to increased reactive ferrous iron [128].

## FTL and neuroferritinopathy

Ferritin is the major storage protein for cellular iron (Figure 1). Twenty‐four monomers consisting of the heavy and light chain subunits form a proteinaceous shell that stores iron. Ferritin can store up to 4500 iron atoms. The heavy chain has a ferroxidase activity, and the light chain aids mineralization within the ferritin structure.

Mutations that affect the ferritin light chain (FTL) lead to the autosomal dominant disease neuroferritinopathy. The most common insertion mutation may lead to a poisoning of the holo‐ferritin structure, potentially at the iron entry pore [129], leading to an iron‐porous ferritin structure [130].

Iron deposition has been described throughout the basal ganglia from MRI studies [131]. Importantly, evidence of iron deposition has been seen in presymptomatic familial carriers of the disease, leading to the hypothesis that iron accumulation begins in childhood and worsens until symptoms begin, in the fourth decade of life [132].

Pathological investigations have shown ferritin inclusions are present in glia and neurones, as well as confirming iron deposition [19, 30, 31]. Neurones and glia exhibit Ferritin inclusions in the putamen, GP, thalamus and cerebellum. In the cortex, inclusion‐positive glia were perivascular and perineural. Aggregates stain positive for ferritin heavy chain, FTL, iron and ubiquitin. Neuroaxonal swellings have been described, displaying ubiquitin reactivity.

Mitochondrial abnormalities have been highlighted, and an increased oxidative stress of the cells, possibly due to iron, was shown via peroxidation and nitrosylation [31]. Indeed, several animal and cell systems have confirmed an increase in oxidative stress in *FTL* mutant models: through mitochondrial and nuclear DNA damage, proteasomal insufficiencies, and damage to proteins and lipid via reactive species [133, 134, 135]. Iron chelators were able to reverse cell sensitivity, promoting iron as the main cause of disease [133].

### Unifying theories

Despite the fact that mutations in a diverse range of genes lead to NBIA, there are several emerging themes that hint of a mechanistic overlap across the NBIA spectrum: mitochondrial involvement, ROS damage, lipid metabolism, iron and autophagy.

### Iron

One major question that remains unanswered is whether iron accumulation is causative or symptomatic of NBIA. Iron accumulation is not specific to NBIA but is observed in a range of neurodegenerative disorders including AD, HD and PD. This excess iron leads to increased oxidative stress and neuronal toxicity.

Cp and FTL have a direct role in the cellular iron homeostatic pathway. It seems rational that mutations in these genes will lead to iron accumulation; for example, mutant Cp leads to increased transferrin‐free iron and uncontrolled cellular uptake [125].

Other NBIA genes seem more obscure with regard to iron homeostasis, but similar neuropathology and gross clinical symptoms could argue that mechanisms are conserved between all NBIA subtypes. Contrary to this is the argument that single gene defects such as *PLA2G6* lead to distinct diseases with variable iron accumulation. Indeed, iron chelation therapy was able to reduce iron deposition on MRI in PKAN patients but did not lead to clinical benefit [136], suggesting that iron accumulation is not causative of symptoms in NBIA. One explanation for this is that iron accumulation could be indirectly associated with disease. Divalent metal ions have similar properties in cells, and iron dysregulation may affect the steady state of other metal ions – such as zinc or copper leading to neurodegeneration [137].

Iron dyshomeostasis could be explained from a faulty mitochondrial component, as many NBIA‐related genes implicate mitochondria. Mitochondria can act as a cellular iron sink with a dedicated mitochondrial ferritin and a series of enzymes requiring iron and haem for function. However, there has been no discovery of a mitochondrial iron exporter. Therefore, it appears the major mechanism for iron recycling is via mitophagy and lysosomal degradation of iron‐containing proteins (Figure 2) [138]. Indeed, techniques for staining iron primarily depict iron in lysosomal compartments [139]. Altered mitochondrial fitness may therefore alter mitophagic rates and so iron turnover.

### Lipid metabolism

Several of the NBIA genes suggest altered lipid metabolism as a potential disease mechanism: *PANK2, COASY, PLA2G6, FA2H* and potentially *C19orf12*. Altered lipid metabolism would affect synthesis and remodelling of lipid bilayers, and Kotzbauer *et al*. hypothesized a mitochondrial specific fatty acid synthase pathway, which could employ these genes for membrane remodelling and mitochondrial function [37].

Distortion of mitochondrial cristae structure is evident in some NBIA mutant conditions, and it is has been theorized that respiratory chain super structures might be disrupted as a result of lipid insufficiencies [41]. Mitochondrial membrane abnormalities could explain the respiratory deficiencies and ROS damage seen in many NBIA subtypes, and gradually lead to neuronal death.

Lipid metabolism may have a central role in myelin production, and alterations could lead to neurodegeneration. FAH2 is involved in ceramide production, and both PANK2 and COASY are required for sphingomyelin production, demonstrated as *PANK2* mutation carriers have decreased sphingosine and cholesterol [50], two critical components of myelin.

### Autophagy and mitophagy

Lipid metabolism can be linked with autophagy‐associated WDR45 and ATP13A2 via the endosome‐autophagolysosome pathways. It is conceivable that genes involved in lipid metabolism are required to construct lipid bilayers for autophagosome vesicle formation. Mitochondria may represent an intersection of lipid metabolism and autophagy. Not only have mitochondria been shown to donate membranes to autophagic vesicles but also autophagy‐associated proteins are transiently seen on mitochondrial outer membranes [103]. Mitochondrial‐ER contact sites may be critical for this process [140], although this theory is still controversial [141].

In yeast, mitochondrial health requires one of either mitochondrial‐ER connections, or mitochondrial‐vacuole connections (the yeast lysosomal orthologue) [142]. These contacts are critical for lipid, calcium and amino acid transfer, yeast health, and metabolic status [143]. This again hints that mitochondria and component recycling are critical for cellular health.

It is tempting to speculate iron‐laden autophagolysosomes can also link into an exocytosis pathway (Figure 2). Mitochondria were recently shown to be exocytosed for astrocytic degradation [144]. This could explain neuronal and glial susceptibility for iron deposition in affected brain regions. For example, defective mitochondria (*PANK2, COASY, PLA2G6, C19orf12*) may be packaged into early autophagosomes via *WDR45. ATP13A2* might load autophagic vesicles rich in mitochondria with excess iron from the cytoplasm. These could then be secreted and recycled in neighbouring astrocytes.

Unconventional modes of exocytosis are used in several mammalian systems, for example, interleukin secretion [145], and yeast unconventional exocytosis requires autophagic vesicles and acyl‐CoAs: yet another role for CoA [146, 147].

### Unfolded protein response

Tau, α‐synuclein, Cp and FTL have all been shown to produce cellular inclusions in NBIA tissue. Taken together with the presence of Ubiquitin in the axonal swellings described in NBIA, the unfolded protein response could be central to cell death in NBIA [148].

Axonal swellings and tubulovesicular structures are another common theme. Components of these pathological hallmarks include mitochondrial components, ubiquitinated proteins and cytoskeletal alterations. Potential mechanisms of cellular disturbance include local sites of oxidative damage, for example, due to axonal transport defects, local mitochondrial ROS production and membrane damage [80, 81].

### Energetics

Finally, the energetic state is implicated throughout NBIA. Mitochondrial health, together with CoA metabolism and lipid homeostasis all point towards the metabolism of fatty acids and amino acids. Insufficiencies of mitochondrial energy production and generation of ROS could point to defective metabolism and subsequent oxidative damage [149]. Acetyl‐CoA concentration and COASY protein complexes have recently been shown to be cellular sensors of energy status and can directly bridge metabolic state and autophagic flux through CoA concentration [48, 49], further linking *PANK2* and *COASY* to *WDR45* and *ATP13A2*.

Overlap between NBIA disorders and other neurodegenerative disorders is seen via neuropathological evidence of tau and synuclein aggregates, through clinical manifestations and also iron dyshomeostasis. It is tempting to consider shared mechanisms between NBIA, PD, FTD and ALS. The fact that NBIA is often child‐onset may highlight specific mechanisms that go awry in late‐onset diseases due to a reduction of putative ‘coping mechanisms’ for natural stressors. Therefore, mechanisms linking NBIA neurodegeneration among subtypes may bring to light important insights into neuronal health and ageing.

## Conclusions

Using rare, penetrant and genetically defined disorders to understand disease mechanisms can help to make more general conclusions. Cellular processes that lead to neurodegeneration may be shared between a range of neurodegenerative diseases, reinforced by similar pathological and clinical findings.

The specific susceptibility of the GP to iron accumulation leading to movement disorders is intriguing due to the ubiquitous expression of the 10 NBIA genes. This may point to a specific high iron content of the GP [2], a reliance on iron‐rich glial support cells or due to the tonic activity of the GP, a very high metabolic demand. Retinal cells also have a high metabolic rate and are often affected in NBIA patients.

The involvement of the cerebellum correlates with disease severity, e.g. PLAN and FAHN. Additionally, the early involvement of the SN (e.g. BPAN) might stratify the NBIA genes into Parkinsonian‐like and dementia‐like categories, helping to explain the Lewy body and tau‐positive and negative NBIA subtypes.

Future experiments will explain why cellular coping mechanisms are devoid in NBIA mutations, and these perturbations will be of huge interest to more common neurodegenerative studies.

## Author contributions

CA drafted the manuscript. AL, HH and SW provided critical reading and final editing.
